# Up-regulation of CIT promotes the growth of colon cancer cells

**DOI:** 10.18632/oncotarget.18615

**Published:** 2017-06-27

**Authors:** Zehua Wu, Xiangying Zhu, Wendi Xu, Yu Zhang, Lin Chen, Fabo Qiu, Binyuan Zhang, Liqun Wu, Zhihai Peng, Huamei Tang

**Affiliations:** ^1^ Department of Hepatobiliary Surgery, The Affiliated Hospital of Qingdao University, Qingdao 266003, People's Republic of China; ^2^ Department of General Surgery, Shanghai Jiaotong University Affiliated First People's Hospital, Shanghai 200080, People's Republic of China; ^3^ R & D Department, Shanghai GeneChem Limited Company, Shanghai 201203, People's Republic of China; ^4^ Department of Continuing Medical Education, The Affiliated Hospital of Qingdao University, Qingdao 266003, People's Republic of China; ^5^ Department of Pathology, Shanghai Jiaotong University Affiliated First People's Hospital, Shanghai 200080, People's Republic of China

**Keywords:** CIT, colon cancer, cell growth, apoptosis, p53

## Abstract

Colon cancer is one of the major causes of cancer mortality worldwide. However, the underlying mechanism and therapeutic targets of colon cancer have not yet been fully elucidated. In the present study, we demonstrate that citron rho-interacting, serine/threonine kinase 21 (CIT) promotes the growth of human colon cancer cells. CIT is overexpressed in human colon cancer tissues and cell lines. High expression of CIT predicts poor survival for patients with colon cancer. In colon cancer cells, CIT knockdown represses cellular proliferation and colony formation. Our *in vivo* xenograft experiments showed that CIT knockdown reduces the growth rate of colon cancer cells and the final tumor weight. We found that CIT knockdown induces cell cycle arrest and apoptosis in colon cancer cells. Further microarray and bioinformatics analyses indicated that CIT regulates the p53 signaling pathway, which may account for the effects of CIT on colon cancer cells. Taken together, our findings provide evidence that CIT may promote the development of colon cancer, at least in part, through the p53 signaling pathway. Therefore, CIT may be a potential therapeutic target for colon cancer treatment.

## INTRODUCTION

Colon cancer is one of the major causes of cancer mortality worldwide. Every year, colon cancer affects 1.23 million people and causes 608,000 deaths worldwide [1, 2]. Globally, the incidence of colon cancer varies considerably and is closely associated with a western lifestyle. Despite strong hereditary components, most cases of colorectal cancer are sporadic and develop slowly over several years through the adenoma-carcinoma sequence [3]. Five-year relative survival is greater than 90% for patients with stage I disease. However, for patients with stage IV disease, survival is only slightly greater than 10% [3, 4]. The underlying mechanism and therapeutic targets of colon cancer have not yet been fully elucidated.

Citron rho-interacting, serine/threonine kinase 21 (CIT) is present at the cleavage furrow and the midbody during cytokinesis. CIT is essential for cellular abscission [5, 6] and can phosphorylate the regulatory light chain of myosin II, which is the primary motor protein responsible for cytokinesis [7, 8]. CIT kinase (CIT-K) controls the molecular network required for midbody formation during cytokinesis [9]. CIT-K regulates cytokinesis at a step after Rho in the contractile process [5] by maintaining RhoA localization at the cleavage site, which is necessary for proper RhoA activity and contractile ring dynamics [10]. Nevertheless, the physiological and pathological role of CIT remains largely unknown. In mammals, the loss of CIT-K leads to massive cytokinesis failure and apoptosis in only neuronal progenitors and male germ cells, resulting in severe microcephaly and testicular hypoplasia [11]. However, the cause of this specificity is unknown. Recent studies indicate that CIT plays a key role in the development of human cancer, including hepatocellular carcinoma [12]. However, the role of CIT in human colon cancer remains unknown.

In the present study, we demonstrate that CIT promotes the growth of human colon cancer cells. We first found that CIT was overexpressed in human colon cancer tissues. Then, we knocked down CIT and found that CIT knockdown inhibited the proliferation and colony formation of colon cancer cells *in vitro* and reduced colon cancer cell growth *in vivo*. We also found that CIT knockdown regulated the cell cycle and apoptosis partly through the p53 pathway. Therefore, our findings indicate that CIT promotes the growth of human colon cancer cells.

## RESULTS

### CIT is overexpressed in human colon cancer tissues and cell lines

**Figure 1:**
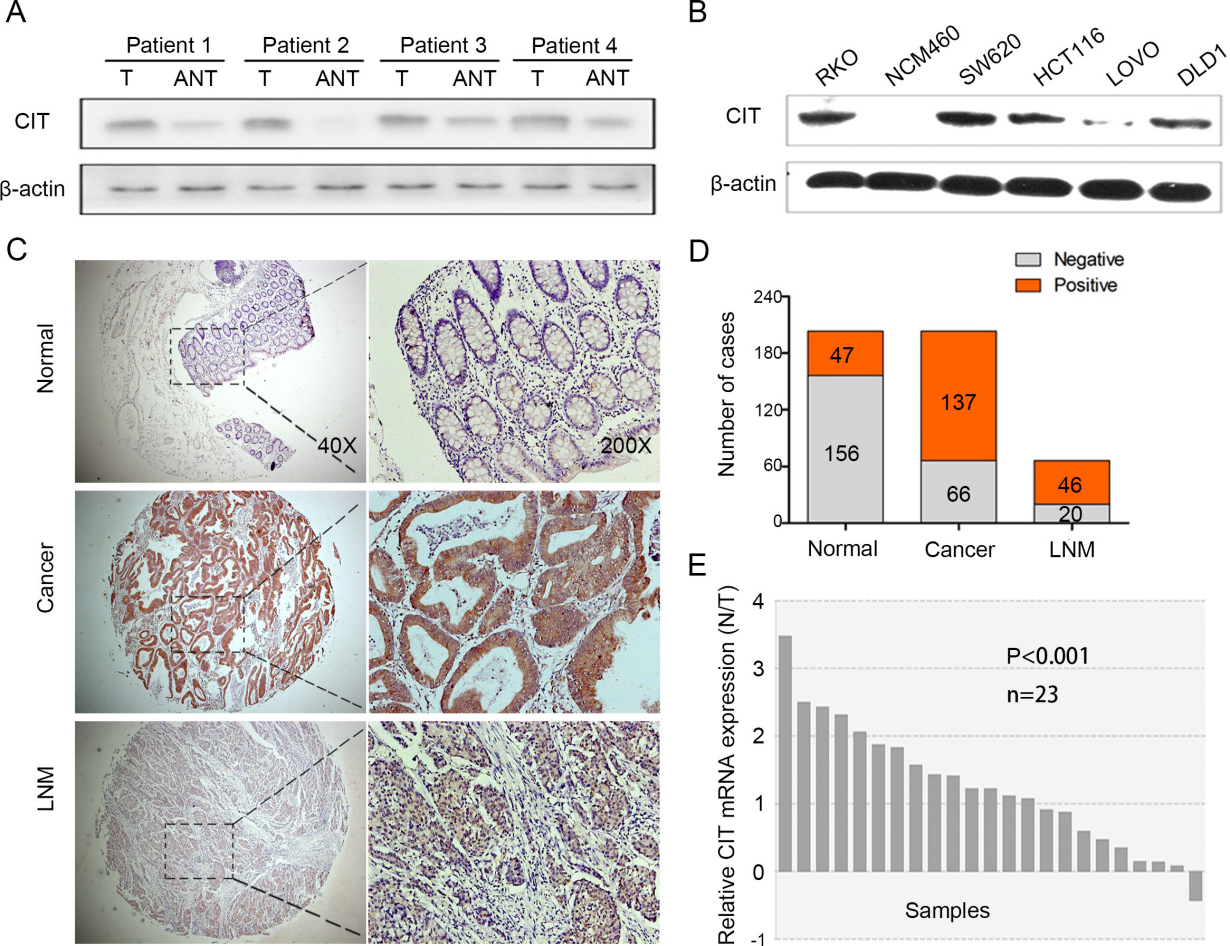
CIT is overexpressed in human colon cancer **(A)** Western blot analysis of CIT in primary colon cancer tissues (T) and matched adjacent noncancerous tissues (ANT). β-Actin was used as a loading control. **(B)** Western blot analysis of CIT in the human colon mucosal epithelial cell line NCM460 and in five colon cancer cell lines. β-Actin was used as a loading control. **(C)** Immunohistochemical (IHC) staining analysis of CIT protein expression in primary colon cancer tissues, matched adjacent noncancerous tissues, and lymph node metastasis specimens. **(D)** A schematic representation of CIT expression from colon cancer tissue microarrays evaluated by IHC staining in **(C)**. **(E)** Relative CIT expression levels in colon cancer tissues and the matched adjacent tissues of the patients included in the Cancer Genome Atlas (TCGA) database (n=23).

To investigate the potential role of CIT in human colon cancer, we first investigated the expression patterns of CIT in human colon cancer tissues and cell lines. Western blotting revealed that CIT protein levels were significantly up-regulated in 4 tested colon cancer tissues compared with matched adjacent normal tissues (Figure 1A). CIT protein levels were up-regulated in five human colon cancer cell lines compared with the human colon mucosal epithelial cell line NCM460 (Figure 1B). Next, we examined CIT expression in 203 pairs of colon cancer tissues and matched adjacent normal tissues, as well as in 66 lymph node metastasis specimens, using immunohistochemical staining. As shown in Figure 1C, CIT was predominantly localized to the cytoplasm. While CIT staining in normal tissues was 23.2% positive, CIT staining in cancer tissues was 67.5% positive, and CIT staining in lymph node metastasis specimens was 69.7% positive (Figure 1C and 1D, P<0.001), indicating that CIT is up-regulated in colon cancer tissues and metastatic specimens. We next analyzed CIT expression in an independent cohort of colon cancer patients using RNA sequencing in 23 colon tumor tissues and matched adjacent normal tissues. As showed in Figure 1E, the CIT gene was significantly higher expressed in the colon tumor tissues compared with the adjacent normal tissues. Among the 23 enrolled samples, CIT expression was elevated in 22 colon tumor tissues. Taken together, these results indicate that CIT is associated with colon tumorigenesis.

### CIT expression is associated with the clinical features of colon cancer

**Figure 2:**
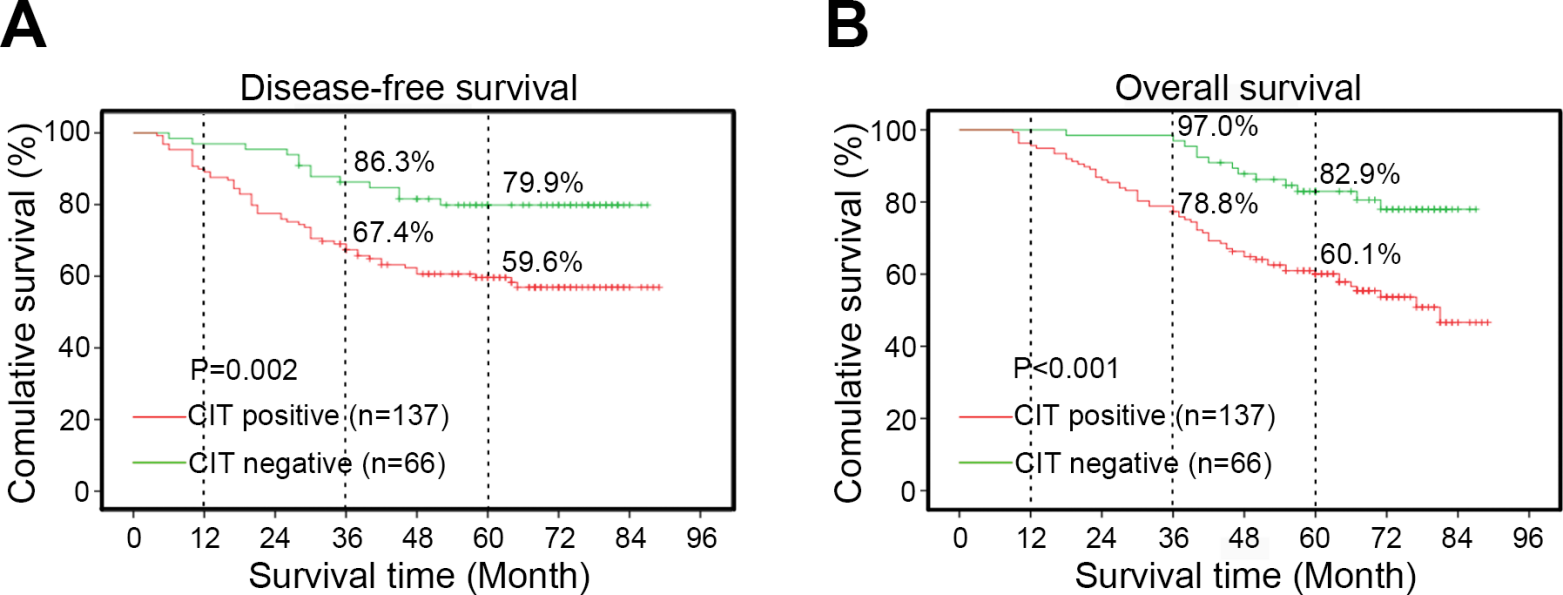
Kaplan-Meier curves based on CIT expression **(A-B)** Disease-free survival (DFS) and overall survival (OS) curves of 203 primary colon tumors. The survival rate of the CIT-positive group (n=137) is significantly lower than that of the CIT-negative group (n=66). DFS P=0.002; OS P<0.001.

Next, we investigated the relationship between CIT expression and clinical features of colon cancer patients. We divided the patients into two groups, a CIT high/positive group (n=137) and a CIT low/negative group (n=66), based on the immunohistochemical staining results shown in Figure 1D. We found that high CIT levels were strongly associated with high AJCC stage (*P*=0.040), T stage (*P*=0.008) and M stage (*P*=0.011, Table 1). Moreover, the Kaplan–Meier survival curves and the log-rank test survival analysis showed that the disease-free survival (DFS) and overall survival (OS) of patients with CIT-positive tumors were significantly poorer than those of patients with CIT-negative tumors (DFS: *P*=0.002, Figure 2A; OS: *P*<0.001, Figure 2B). These results suggest that CIT expression is associated with poor survival in patients with colon cancer.

**Table 1:** CIT expression and clinicopathologic characteristics

	CIT expression		*P* Value
	Negative (n=66, %)	Positive (n=137, %)	
**Age**			0.308
<65	23 (28.4)	58 (71.6)	
≥65	43 (35.2)	79 (64.8)	
**Gender**			0.126
Male	33 (38.4)	53 (61.6)	
Female	33 (28.2)	84 (71.8)	
**Location**			0.607
Right	29 (34.5)	55 (65.5)	
Others	37 (31.1)	82 (68.9)	
**AJCC Stage**			0.040*
I + II	41 (39.0)	64 (61.0)	
III + IV	25 (25.2)	73 (74.5)	
**T Stage**			0.008*
T_1_ + T_2_	16 (53.3)	14 (46.7)	
T_3_ + T_4_	50 (28.9)	123 (71.1)	
**N Stage**			0.077
N_0_	41 (38.0)	67 (62.0)	
N_1_ + N_2_	25 (26.3)	70 (73.7)	
**M Stage**			0.011*
M_0_	65 (35.1)	120 (64.9)	
M_1_	1 (5.6)	17 (94.4)	
**Differentiation**			0.149
Well + Moderate	37 (37.4)	62 (62.6)	
Poor	29 (27.9)	75 (72.1)	
**Vascular invasion**			0.131
Yes	64 (33.9)	125 (66.1)	
No	2 (14.3)	12 (85.7)	

### CIT promotes colon cancer cell growth *in vitro*

**Figure 3:**
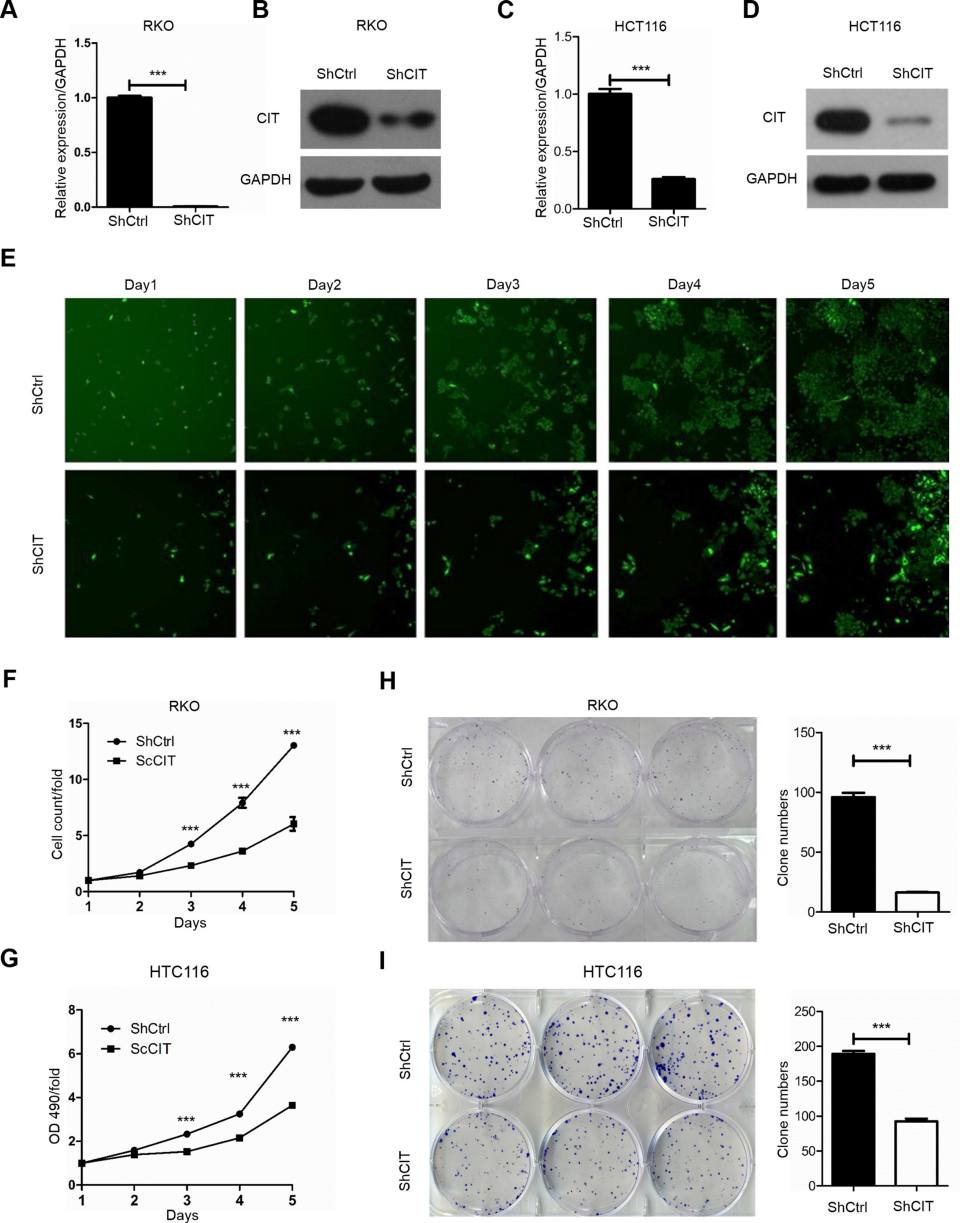
Knockdown of CIT inhibits human colon cancer cell growth *in vitro* **(A-B)** Quantitative RT-PCR and western blot analysis show that CIT expression was efficiently knocked down in the RKO cells. **(C-D)** Quantitative RT-PCR and western blot analysis show that CIT expression was efficiently knocked down in the HCT116 cells. **(E)** Representative pictures of RKO cells infected with Control-shRNA (top) and CIT-shRNA (bottom) via multiparametric high-content screening (HCS) every day for five days. **(F)** CIT knockdown inhibits the proliferation of RKO cells. ***P<0.001 vs. sh-Ctrl. **(G)** CIT knockdown inhibits the proliferation of HCT116 cells. ***P<0.001 vs. sh-Ctrl. **(H-I)** Colony formation assay of RKO and HCT116 cells after infection with sh-Ctrl and sh-CIT lentivirus. ***P<0.001 vs. sh-Ctrl.

To investigate the role of CIT in human colon cancer, we knocked down CIT with lentivirus-mediated shRNA in RKO and HCT116 cell lines. CIT expression was significantly reduced by sh-CIT in RKO and HCT116 cells at both the mRNA and protein levels (Figure 3A-3D). To study the effects of CIT on cell proliferation, RKO cells were infected with sh-Ctrl or sh-CIT lentivirus, and cell growth was monitored by high-content screening (HCS) every day for five days. As shown in Figure 3E and 3F, CIT knockdown significantly suppressed cell growth, and similar results were detected in HCT116 cells using an MTT assay (Figure 3G). Moreover, we analyzed colony formation to determine the contribution of CIT to colon cancer cell tumorigenesis *in vitro*. The results showed that CIT knockdown in both RKO and HCT116 cells dramatically reduced colony formation (Figure 3H and 3I). These results show that CIT is necessary for colon cancer cell proliferation and colony formation *in vitro*.

### CIT promotes colon cancer cell growth *in vivo*

**Figure 4:**
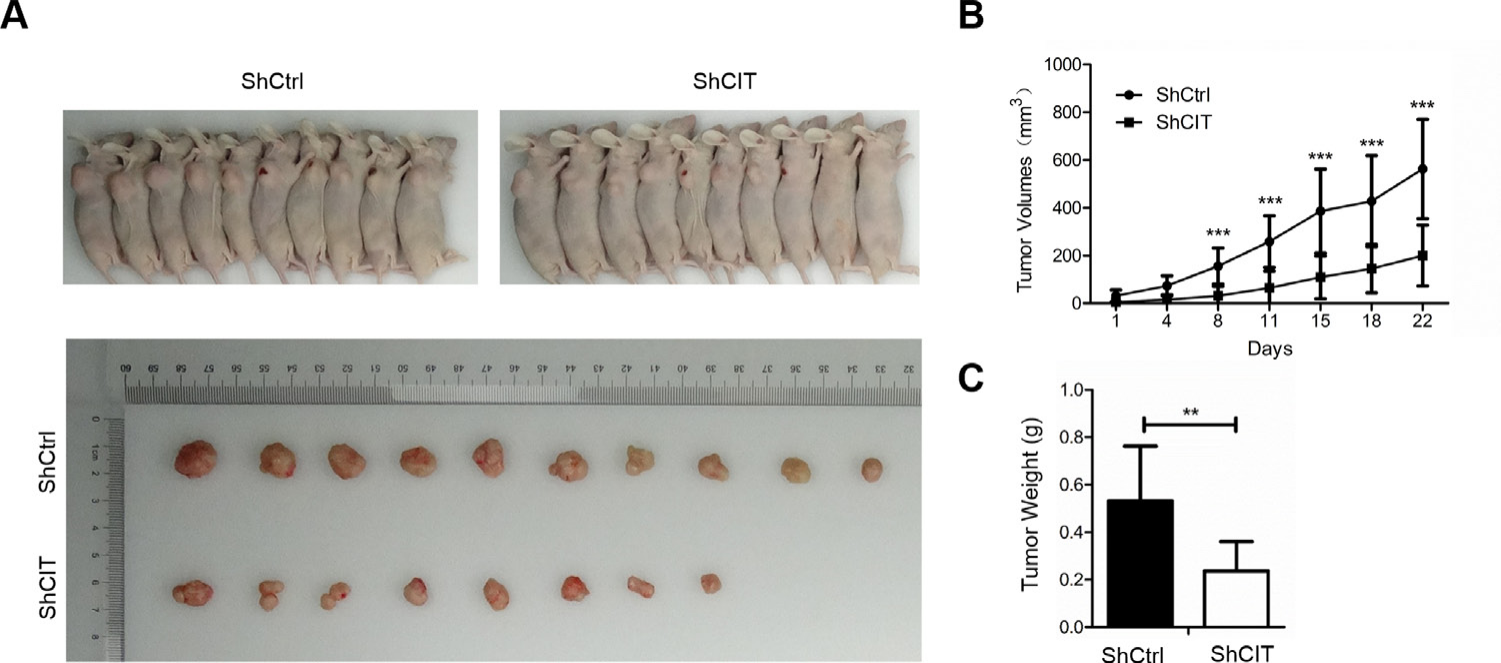
CIT knockdown represses colon cancer cell growth *in vivo* **(A)** Subcutaneous tumors in nude mice and isolated tumors after 4 weeks formed by injection of RKO cells infected with sh-Ctrl and sh-CIT lentivirus (n=10 in each group). **(B)** Tumor growth curves of xenografts in nude mice (n=10 in each group). Xenograft volumes were calculated using the formula v=0.5ab^2^ (a: long diameter, b: short diameter). ***P<0.001 vs. sh-Ctrl. **(C)** Statistics for the tumor weights of the xenografts (n=10 in each group). **P<0.01 vs. Sh-Ctrl.

To investigate the function of CIT in colon cancer cell growth *in vivo*, we evaluated xenograft formation of HCT116 cells with/without stable CIT knockdown. As expected, after four weeks of injection, the tumors in the CIT knockdown group were significantly smaller than those in the control group (Figure 4A and 4B). Additionally, tumor growth curves were constructed based on the measurements of tumor volumes of the xenografts for 28 consecutive days. This analysis revealed that CIT knockdown significantly reduced tumor volume (Figure 4C). Taken together, these data indicate that CIT promotes the xenograft growth of colon cancer cells.

### Knockdown of CIT induces cell cycle arrest and apoptosis

**Figure 5:**
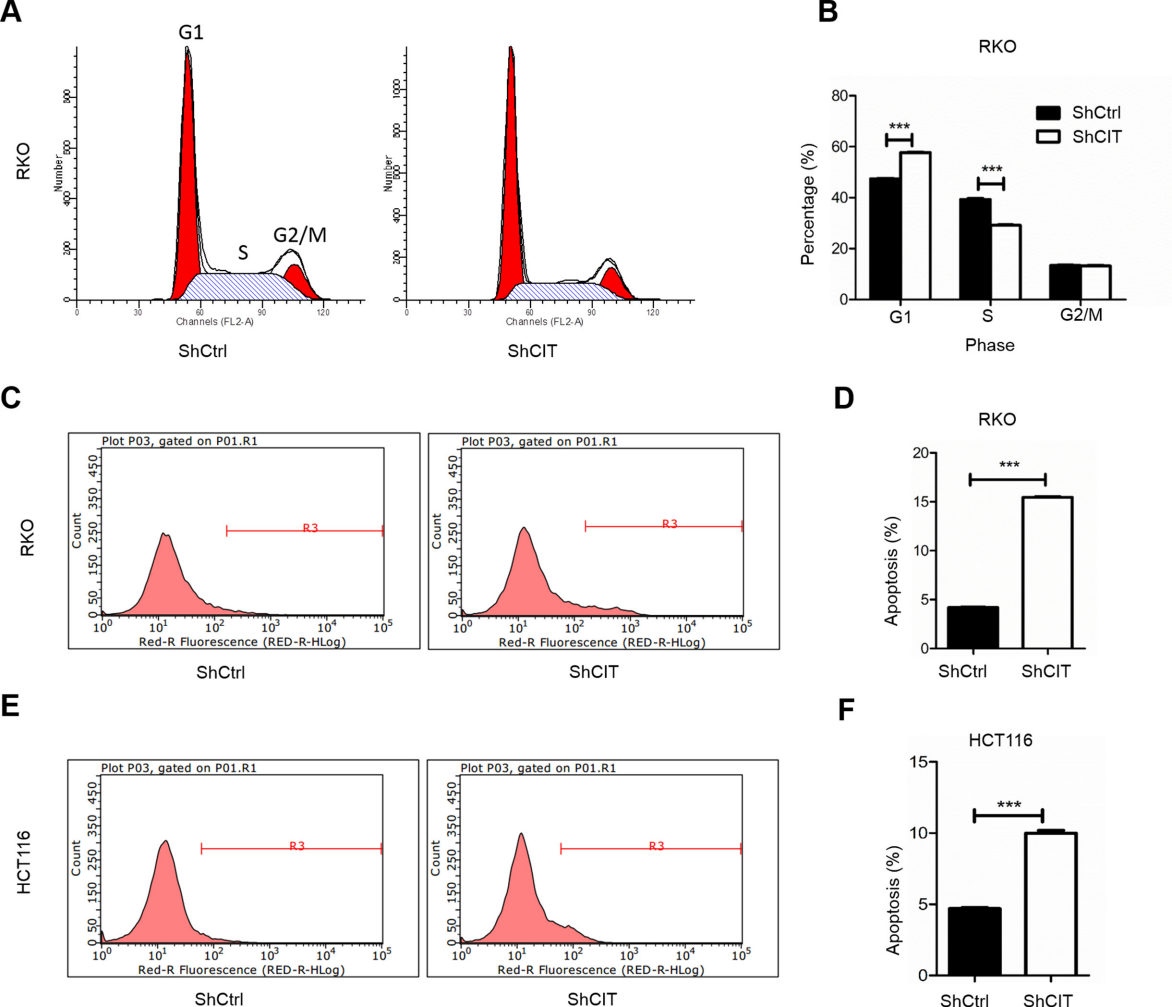
Knockdown of CIT induces cell cycle arrest and apoptosis **(A-B)** Flow cytometry analysis of the cell cycle reveals that CIT knockdown induces RKO cell cycle arrest at the G1 phase (G1 phase: 47.34% ± 0.52% vs. 57.72% ± 0.40%; S phase: 39.28% ± 0.78% vs. 29.14% ± 0.34%; and G2/M phase: 13.38% ± 0.31% vs. 13.14% ± 0.53%). ***P<0.001 vs. sh-Ctrl. **(C-D)** Flow cytometry analysis of apoptosis reveals that the rates of apoptosis of RKO cells are significantly increased by CIT knockdown (5.02% ± 0.29% vs. 19.60% ± 0.29%). ***P<0.001 vs. sh-Ctrl. **(E-F)** Flow cytometry analysis of apoptosis reveals that the rates of apoptosis of HCT116 cells are significantly increased by CIT knockdown (4.70% ± 0.13 vs. 9.99% ± 0.34%). ***P<0.001 vs. sh-Ctrl.

Cell cycle arrest and apoptosis usually have an effect on cell proliferation. To determine whether CIT inhibits colon cancer cell proliferation by regulating the cell cycle and apoptosis, we examined the cell cycle by PI/FACS and detected apoptosis by fluorescence activated cell sorting (FACS). As shown in Figure 5A and 5B, knockdown of CIT expression led to cell cycle arrest in the G0/G1 phase (47.3% for sh-Ctrl vs. 57.7% for sh-CIT). Additionally, CIT knockdown induced cell cycle arrest in HCT116 cells (Supplementary Figure 1). Furthermore, the percentage of apoptotic RKO cells was significantly increased in the CIT knockdown group compared with the control group (Figure 5C and 5D, 4.35% for sh-Ctrl vs. 15.46% for sh-CIT), and similar results were observed in HCT116 cells (Figure 5E and 5F). These results indicate that CIT regulates the cell cycle and apoptosis of colon cancer cells.

### Knockdown of CIT suppresses the p53 and apoptosis pathways

**Figure 6:**
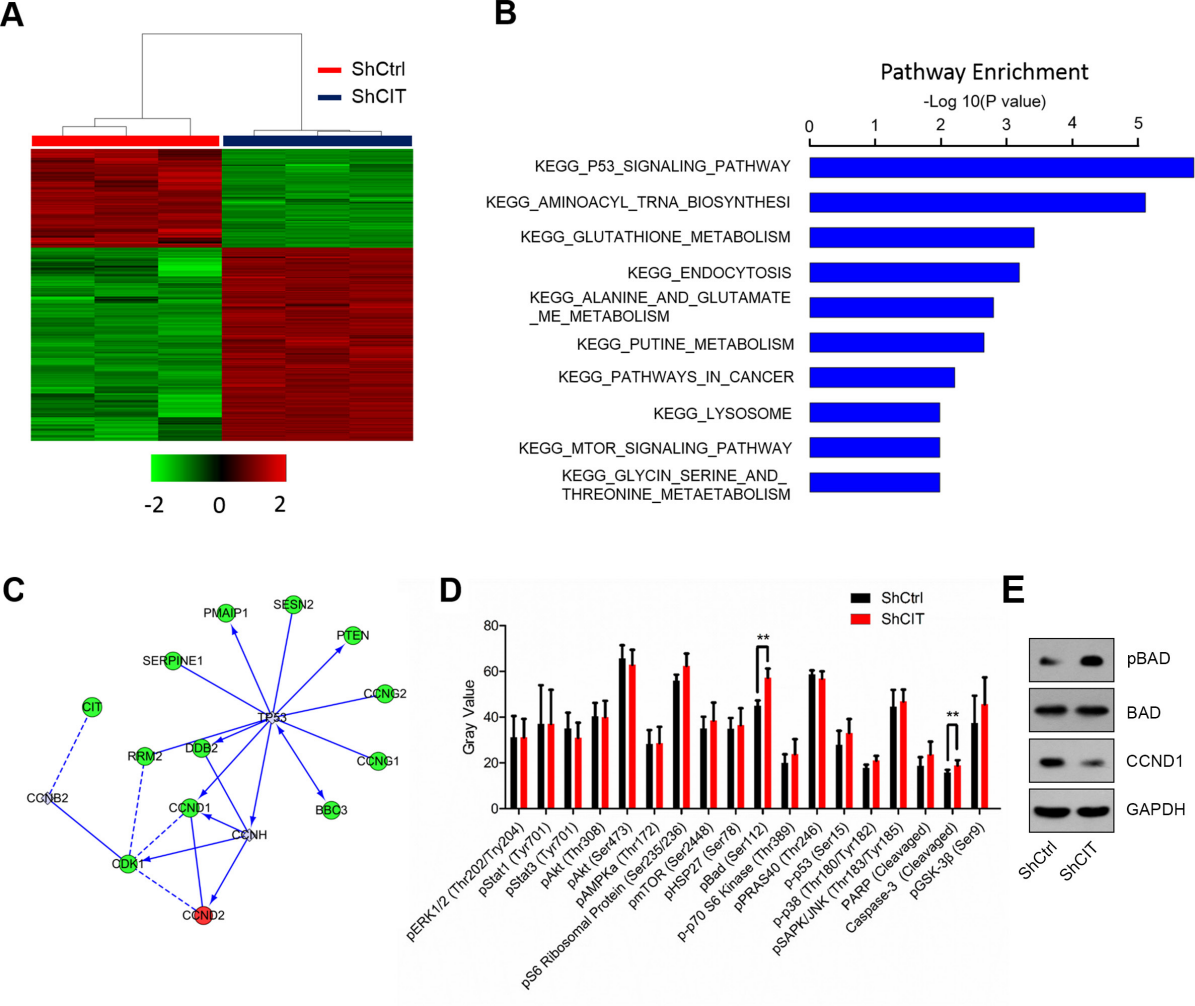
Down-regulation of CIT affects the p53 and apoptosis pathways **(A)** Heat map of 885 genes (295 genes up-regulated and 590 genes down-regulated) showing significant differential expression patterns in RKO cells infected with sh-Ctrl or sh-CIT lentivirus (criteria: *P*<0.05 and absolute fold change >1.5). **(B)** Functional pathway enrichment of differential genes analyzed based on the KEGG and BIOCARTA databases. The P53 signaling pathway was the most significantly enriched pathway (*P*=1.44E-06). **(C)** Interaction network between CIT and the genes involved in the KEGG pathway P53. Green circles represent down-regulated genes, red circles represent up-regulated genes, and gray circles represent genes with no change in expression. **(D)** The post-translational modification of 18 important signaling proteins (16 phosphorylation proteins and 2 cleavage proteins) was monitored by array hybridization. CIT knockdown increases Bad phosphorylation and Caspase-3 cleavage in RKO cells. **P<0.01 vs. sh-Ctrl. **(E)** CIT knockdown regulates Bad and CCND1. RKO cells were infected with sh-CIT or sh-Ctrl for 48 h. Then, cell lysates were subjected to western blotting with the indicated antibodies. GAPDH was used as an internal control.

The above results showed that CIT was required for colon cancer cell growth *in vitro* and *in vivo*, partly by regulating colon cancer cell cycle and survival. However, the mechanisms underlying CIT-mediated phenotypes in colon cancer cells were still unknown. To address this, global gene expression profiling was analyzed in RKO cells with/without CIT knockdown (Figure 5A). We found that 295 genes were up-regulated and that 590 genes were down-regulated by CIT knockdown (Figure 6A). Next, we performed a KEGG pathway analysis and found that the differentially expressed genes were enriched in ten pathways (Figure 6B). The p53 signaling pathway was the most significantly enriched pathway in cells with CIT knockdown (*P*=1.44E-06, Figure 6B). Next, we analyzed the functional interaction network between CIT and the p53 signaling pathway. As shown in Figure 6C, cell cycle regulators (CCND1, CCNG1, CCNG2, CDK1, SESN2 and RRM2) of the p53 signaling pathway were down-regulated by CIT knockdown.

To further understand the intracellular signaling pathway induced by CIT in colon cancer cells, we analyzed key proteins involved in cell proliferation, cell growth, cell cycle regulation and apoptosis using the PathScan^®^ intracellular signaling array kit. Interestingly, CIT knockdown could significantly promote the phosphorylation of Bad and the cleavage of caspase-3 (Figure 6D). Finally, we validated by western blotting that CIT knockdown up-regulated p-Bad and down-regulated CCND1 in RKO cells (Figure 6E). These results indicate that CIT knockdown suppressed colon cancer tumorigenesis via the p53 and apoptosis pathways.

## DISCUSSION

In the present study, we demonstrate that CIT acts as a tumor promoter in human colon cancer. CIT expression is up-regulated in human colon cancer tissues, and up-regulation of CIT is associated with advanced disease stage and poor survival. Using loss-of-function experiments, we show that CIT knockdown inhibits proliferation and colony formation of colon cancer cells *in vitro* and growth of colon cancer cells *in vivo*. Our investigation of the mechanism indicates that CIT knockdown induces cell cycle arrest and apoptosis in colon cancer cells. The microarray data and bioinformatics analysis show that CIT regulates the p53 pathway, which may account for the effects of CIT on colon cancer cells.

Fu *et al*. [12] reported that CIT is frequently up-regulated in human hepatocellular carcinoma compared with adjacent non-tumor tissues. Another study showed that the down-regulation of miR-486 mediates the up-regulation of CIT in hepatocellular carcinoma [15]. In this study, we show that CIT expression is markedly up-regulated in human colon cancer, indicating that CIT may facilitate the development of colon cancer. Yan *et al*. [16] identified and analyzed the epigenetically silenced microRNAs in colorectal cancer cells. They found that miR-1247, which is predicted to target CIT (http://www.microrna.org), was significantly silenced in colon cancer cells [16]. Therefore, the lack of miR-1247 may result in increased CIT expression in colon cancer tissues. However, further experimental evidence is needed to confirm this.

We found that CIT knockdown inhibits proliferation and colony formation in the colon cancer cell lines RKO and HCT116. Additionally, our xenograft experiments revealed that CIT knockdown repressed colon cancer cell growth *in vivo*. CIT is present at the cleavage furrow and midbody during cytokinesis and is essential for cellular abscission. CIT also regulates the G2/M transition in rat hepatocytes, and knocking down CIT inhibits the proliferation of hepatocellular carcinoma cells [12]. We found that CIT knockdown also induced cell cycle arrest in colon cancer cells. However, while CIT knockdown inhibits the G1/S transition in colon cancer cells, CIT knockdown does not inhibit the G2/M transition as is observed in hepatocellular carcinoma cells. Furthermore, the percentage of apoptotic cells was significantly up-regulated when CIT was knocked down in colon cancer cells. These findings indicate that CIT regulates the cell cycle and cell survival to promote the development of colon cancer.

The tumor suppressor p53 is a transcription factor that regulates several genes with a broad range of functions, including DNA repair, metabolism, cell cycle arrest, apoptosis and senescence [17, 18]. Many different types of cancer are associated with a high incidence of TP53 mutations, leading to the expression of mutant p53 proteins [19]. There is growing evidence that these mutant p53 proteins have lost wild-type p53 tumor suppressor activity [20, 21]. Alterations in the p53 pathway play a significant role in colon cancer [22]. Interestingly, our microarray and bioinformatics analyses show that CIT knockdown in RKO cells activates genes in the p53 pathway, indicating that p53 signaling plays a pivotal role in the effects of CIT on the cell cycle and apoptosis, which subsequently regulate cell growth and cancer development, of colon cancer cells.

In summary, we provide evidence that CIT is overexpressed in human colon cancer tissues and serves as a tumor promoter. CIT maintains the cell cycle and survival partly by regulating the p53 signaling pathway. Therefore, CIT may be a potential therapeutic target for treating human colon cancer.

## MATERIALS AND METHODS

### Patient information

All 203 patients with colon cancer, including 86 men and 117 women, were enrolled from 2001 to 2003. The median age of the patients was 68 years (range: 22-95) at the time of surgery, and the median follow-up time was 61 months post-operatively (range: 9-89 months). The adjacent normal tissues were taken from an area more than 10 cm away from the primary neoplasms, and paired lymph node metastasized specimens were taken from 66 patients. The study was approved by the Ethics Committee of Shanghai Jiaotong University affiliated with the First People's Hospital. Written informed consent was obtained from all patients.

### Colon cancer mRNA database and analysis

Transcriptome expression datasets and the corresponding clinical information were downloaded from The Cancer Genome Atlas (http://cancergenome.nih.gov). A total of 23 samples, which included transcriptional expression data for both tumor tissues and adjacent normal tissues, were available for this analysis.

### Cell lines and cell culture

The human colon mucosa cell line NCM460 was obtained from INCELL (San Antonio, USA). The human colon cancer cell lines RKO, DLD-1, SW620, LoVo and HCT116 were purchased from the Type Culture Collection of the Chinese Academy of Science (Shanghai, China). Cells were cultured in Dulbecco's modified Eagle's Medium (DMEM)/F12 (Gibco, Invitrogen Corp., San Diego, California, USA) combined with 10% fetal bovine serum and 1% penicillin/streptomycin at 37°C in a humidified atmosphere of 5% CO_2_ and 95% air.

### RNA Isolation and quantitative real-time PCR

Total RNA was extracted and purified using Trizol reagent (Invitrogen) according to the manufacturer's instructions. Reverse transcription was performed using M-MLV reverse transcriptase (Promega) and random primers (Sangon, Shanghai, China) to obtain cDNAs. CIT mRNA expression was analyzed by quantitative real-time PCR using SYBR master mix (Takara) on a real-time PCR machine TP800 (Takara). The sequences of the primers used are as follows: CIT forward, 5′-CAGGCAAGATTGAGAACG-3′; CIT reverse, 5′-GCACGATTGAGACAGGGA-3′; GAPDH forward, 5′-TGACTTCAACAGCGACACCCA-3′; and GAPDH reverse, 5′-CACCCTGTTGCTGTAGCCAAA-3. The relative CIT expression was normalized to GAPDH, and data analysis was conducted using the comparative CT method.

### Western blot

Cells were washed with PBS and lysed with lysis buffer (Beyotime). Total protein levels were measured using a BCA protein assay kit (Pierce). Western blotting was performed as described previously [13]. Briefly, 20 μg of total protein was mixed with 2× loading buffer and separated by 10% sodium dodecyl sulfate polyacrylamide gel electrophoresis (SDS-PAGE). The proteins were then transferred to polyvinylidene fluoride (PVDF) membranes (Amersham). The PVDF membranes were blocked with 5% fat-free milk dissolved in Tris-buffered saline and Tween 20 (TBST) buffer for 1 h and then incubated with the primary antibodies (anti-CIT, Abcam, ab110897; anti-CCND1, Abcam, ab16663; anti-pBad, Cell Signaling Technology (CST), #9664; anti-Bad, Abcam, ab32445; and anti-GAPDH, Santa Cruz Biotechnology, sc-32233) overnight at 4°C. After three washes with TBST buffer, the secondary antibody (Santa Cruz Biotechnology, sc-2005) was added, and immune activity was detected using an ECL-Plus kit (Amersham Biosciences).

### Tissue microarray construction and immunohistochemistry

Tissue microarrays of 203 pairs of colon cancer and adjacent tissues, as well as 66 lymph node metastases, were performed using immunohistochemical staining. CIT immunoreactivity was assessed independently by two expert pathologists blinded to the clinical data, and the scoring standard used has been described previously [14].

The staining intensity and extent of CIT expression were graded as follows: negative= score 0, weak= score 1, moderate= score 2, and strong= score 3. The extent of staining was categorized by the percentage of strongly stained cells in the cancer nest as follows: negative= score 0, 1 to 25%= score 1, 26 to 50%= score 2, 51 to 75%= score 3 and 76 to 100%= score 4. CIT scoring was assigned to each tumor by multiplying the staining intensity score and the staining extent score. Negative staining refers to a CIT score ≤6, and positive staining refers to a CIT score >6.

### Packaging of sh-CIT lentivirus

The lentivirus vector system included the following vectors: pGCSIL-GFP, which stably expresses a short hairpin RNA (shRNA) and a marker (GFP fusion protein); pHelper1.0 (gag/pol element); and Helper2.0 (VSVG element). The shRNA targeting human CIT (5′-GCGTCCTCATACCAGGATAAA-3′) and the negative control (NC) shRNA (5′- TTCTCCGAACGTGTCACGT-3′) were designed, synthesized and cloned into the pGCSIL-GFP vector by the GeneChem Corporation (Shanghai, China). The pGCSIL–shRNA-GFP, pHelper1.0 and Helper2.0 were mixed and transfected into 293T cells using Lipofectamine TM 2000 (Invitrogen, Shanghai, China) according to the manufacturer's instructions. After transfection for 48 h, the viral supernatants were collected, centrifuged, and then filtered through a 0.45 μm polyvinylidene fluoride membrane.

### High-content screening cell proliferation assay

Cell growth was measured via multiparametric high-content screening (HCS). After 72 h of infection with the NC or CIT-shRNA lentivirus, RKO cells were harvested, re-suspended, counted and inoculated into 96-well plates at 37°C with 5% CO_2_ for 5 days. The proliferation of the cells in each of the wells was analyzed daily using ArrayScan™ HCS software (Cellomics Inc.). The system uses a computerized, automated fluorescence imaging microscope that automatically identifies stained cells and measures the intensity and distribution of fluorescence in each individual cell. Images were acquired for each fluorescence channel using suitable filters and a 20 × objective. Images and data were stored in a Microsoft SQL database.

### MTT cell proliferation assay

Cells infected with the NC or CIT-shRNA lentivirus were seeded in 96-well plates at a density of 2×10^3^ cells/well and incubated at 37°C for 1, 2, 3, 4, or 5 days. Then, the cells were washed two times with PBS, and 3-(4,5-dimethyl-2-yl)-2,5-diphenyltetrazolium bromide (MTT) solution (5 mg/mL) was added to each well. After 4 h of incubation, the supernatants were removed, and then 100 μL of dimethyl sulfoxide (DMSO) was added to solubilize the formazan salt. Ten minutes later, the optical density (OD) was measured at 490 nm using a microplate reader.

### Colony formation assay

Cells infected with the NC or CIT-shRNA lentivirus were seeded in six-well plates (800 cells/well). The media was replaced every 2 to 3 days. After incubation at 37°C for 14 days, adherent cells were washed twice with PBS and then fixed with 4% paraformaldehyde for 30 minutes. The colonies were stained with Giemsa solution for 10 min and then washed twice with double distilled water. The number of colonies (> 50 cells/colony) were counted using a fluorescence microscope (CKX41, Olympus, Tokyo, Japan).

### Apoptosis assay

Apoptosis was analyzed by staining with Annexin V-APC (eBioscience) according to the manufacturer's protocol. Cells were infected with the NC or CIT-shRNA lentivirus. After incubation for another 4 days, the cells were harvested, washed with PBS, and resuspended using staining buffer at a final density of 1×10^6^−1×10^7^/ml. Next, 5 μl annexin V-APC was added to 100 μl cell suspensions Then, the cell suspensions were incubated at room temperature for 15 min and subjected to flow cytometry (FACSCalibur, Becton-Dickinson, USA).

### Cell cycle assay

Cells were infected with the NC or CIT-shRNA lentivirus. After incubation for 96 h, cells were seeded in six-well culture plates and cultured to 80% confluence. Cells were fixed with 70% cold ethanol at 4°C overnight, washed twice with ice-cold PBS, and incubated with 10 mg/ml RNase at 37°C. The cell cycle was monitored by propidium iodide (PI) staining of the nucleus. The fluorescence of DNA-bound PI in cells was measured by flow cytometry (FACSCalibur, Becton Dickinson). The dot plots and gating strategy of FACS analyses were showed in Supplementary Figure 2.

### *In vivo* xenograft assay

Four-week-old male nude mice were used for the xenograft growth curve assay. A total of 4×10^6^ HCT116 cells were subcutaneously injected into the right armpits of the mice, and the xenograft diameters were measured using a slide caliper rule every other day for 28 days. The xenograft tumor volume was calculated using the following formula: v=0.5ab^2^ (where a= long diameter of the tumor, b= short diameter of the tumor, and v= volume).

### Microarray

Total RNA from RKO cells infected with the NC (n=3) or sh-CIT lentivirus (n=3) was extracted using Trizol reagent. A NanoDrop 2000 and an Agilent Bioanalyzer 2100 were used to detect the RNA quantity and quality, respectively. Microarrays were processed to generate gene expression profiles using the Affymetrix human GeneChip PrimeView assay according to the manufacturer's instructions. Genes that were differentially expressed between RKO cells treated with sh-Ctrl and those treated with sh-CIT were identified based on the following criteria: P <0.05 and an absolute fold change >1.5. Pathway enrichment analyses using the KEGG and BIOCARTA databases were performed for all significantly differentially expressed genes.

### Intracellular signaling assay

To determine the intracellular signaling pathway that is responsible for the phenotype induced by CIT, we detected modifications in a set of cellular proteins that play known roles in cell proliferation and apoptosis using a PathScan^®^ intracellular signaling array kit (CST, #7323). RKO cell lysates were prepared and analyzed according to the protocol provided by CST. Each experiment was repeated three times.

### Statistical analysis

The statistical analyses were performed using SPSS version 16.0 (SPSS, Inc., Chicago, IL). The raw data are presented as the mean ± standard error of mean (SEM) of at least three independent experiments, unless otherwise stated. Student's *t* tests were applied to analyze the differences between two groups. Differences among groups were determined by one-way or two-way analysis of variance (ANOVA) with repeated measures, followed by the Bonferroni post hoc test. A P value of less than 0.05 was considered significant.

## SUPPLEMENTARY FIGURES


