# Supplementary material for: Up-regulation of CIT promotes the growth of colon cancer cells
Source: Oncotarget. 2017 Jun 27;8(42):71954–64. doi: 10.18632/oncotarget.18615 (PMC5641103; doi:10.18632/oncotarget.18615)
Supplement: Supplementary file 1 [file oncotarget-08-71954-s001.pdf]

Up-regulation of CIT promotes the growth of colon cancer cells

SUPPLEMENTARY MATERIALS

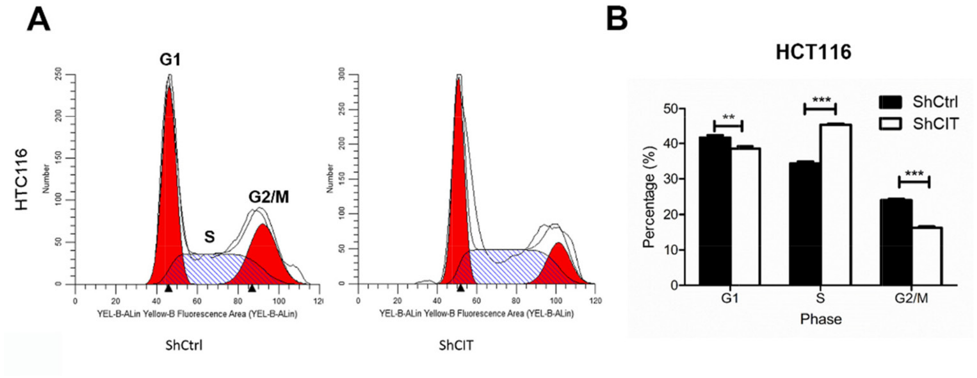

Supplementary Figure 1: (A-B) Flow cytometry analysis of cell cycle reveals that CIT knockdown induces HCT116 cell cycle arrested at S phase. ***P<0.001 vs. sh-Ctrl.

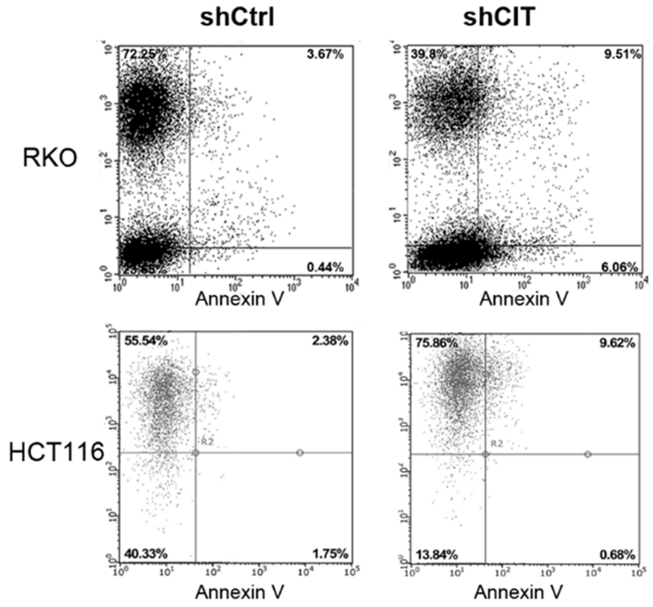

Supplementary Figure 2: This figure showed the dot plots and gating strategy of FACS analyses for Figure 5C & 5E.
